# The alterations in molecular markers and signaling pathways in chronic thromboembolic pulmonary hypertension, a study with transcriptome sequencing and bioinformatic analysis

**DOI:** 10.3389/fcvm.2022.961305

**Published:** 2022-07-26

**Authors:** Wenqing Xu, Mei Deng, Xiapei Meng, Xuebiao Sun, Xincao Tao, Dingyi Wang, Shuai Zhang, Yanan Zhen, Xiaopeng Liu, Min Liu

**Affiliations:** ^1^Department of Radiology, Peking University China-Japan Friendship School of Clinical Medicine, Beijing, China; ^2^Department of Radiology, Chinese Academy of Medical Sciences and Peking Union Medical College, Beijing, China; ^3^Department of Pulmonary and Critical Care Medicine, China-Japan Friendship Hospital, Beijing, China; ^4^Institute of Medicine, China-Japan Friendship Hospital, Beijing, China; ^5^Department of Cardiovascular Surgery, China-Japan Friendship Hospital, Beijing, China; ^6^Department of Radiology, China-Japan Friendship Hospital, Beijing, China

**Keywords:** chronic thromboembolic pulmonary hypertension, differentially expressed lncRNAs, differentially expressed mRNAs, ceRNA network, transcriptome sequencing

## Abstract

**Background:**

At present, the alterations in molecular markers and signaling pathways in chronic thromboembolic pulmonary hypertension (CTEPH) remain unclear. We aimed to compare the difference of molecular markers and signaling pathways in patients with CTEPH and healthy people with transcriptome sequencing and bioinformatic analysis.

**Methods:**

We prospectively included 26 patients with CTEPH and 35 sex- and age-matched healthy volunteers as control. We extracted RNA from whole blood samples to construct the library. Then, qualified libraries were sequenced using PE100 strategy on BGIseq platform. Subsequently, the DESeq2 package in R was used to screen differentially expressed mRNAs (DEmRNAs) and differentially expressed long non-coding RNAs (DElncRNAs) of 7 patients with CTEPH and 5 healthy volunteers. Afterwards, we performed functional enrichment and protein–protein interaction analysis of DEmRNAs. We also performed lncRNA-mRNA co-expression analysis and lncRNA-miRNA-mRNA network construction. In addition, we performed diagnostic analysis on the GSE130391 dataset. Finally, we performed reverse transcription polymerase chain reaction (RT-PCR) of genes in 19 patients with CTEPH and 30 healthy volunteers.

**Results:**

Gender and age between patients with CTEPH and healthy controls, between sequencing group and *in vitro* validation group, were comparable. A total of 437 DEmRNAs and 192 DElncRNAs were obtained. Subsequently, 205 pairs of interacting DEmRNAs and 232 pairs of lncRNA-mRNA relationship were obtained. DEmRNAs were significantly enriched in chemokine signaling pathway, metabolic pathways, arachidonic acid metabolism, and MAPK signaling pathway. Only one regulation pathway of SOBP-hsa-miR-320b-LINC00472 was found through ceRNA network construction. In diagnostic analysis, the area under curve (AUC) values of LINC00472, PIK3R6, SCN3A, and TCL6, respectively, were 0.964, 0.893, 0.750, and 0.732.

**Conclusion:**

The identification of alterations in molecules and pathways may provide further research directions on pathogenesis of CTEPH. Additionally, LINC00472, PIK3R6, SCN3A, and TCL6 may act as the potential gene markers in CTEPH.

## Introduction

Chronic thromboembolic pulmonary hypertension (CTEPH) is classified into Group 4 of pulmonary hypertension (PH) and it is thought to be a severe complication of pulmonary thromboembolism (1). It is characterized by incomplete or abnormal resolution of acute pulmonary embolism, chronic thrombotic mechanical obstruction, pulmonary vascular remodeling, and then right ventricular dysfunction (2, 3). At present, the exact epidemiology and precise pathogenesis of CTEPH are unclear. Therefore, studying the expression of CTEPH-related genes and their interactions is important for understanding the molecular mechanism and providing a potential basis for the further research on pathogenesis of CTEPH. Previous studies (4) indicated that a variety of genes were involved in the progress of CTEPH. Forkhead box class O transcription factor 1 (FoxO1) expression is downregulated in CTEPH, which promotes endothelial cell proliferation and leads to vascular remodeling (4). Chemokine CXC ligand 13 (CXCL13) is overexpressed in pulmonary vascular lesions of patients with CTEPH and has a potential pathogenic role (5). Moreover, abnormal expression of the long non-coding RNAs (lncRNAs) plays an essential role in the progression of CTEPH (6). For example, lncRNA NONHSAT073641 expression is upregulated in CTEPH, which is associated with endothelial angiogenesis (7). LncRNA CTEPH-associated 1 can play an important mediation mechanism in the progress of CTEPH by regulating related networks (8). In addition, microRNAs (miRNAs) also play an important role in the development of CTEPH (9, 10). Such hsa-miR-1226-3p may form competing endogenous RNA (ceRNA) relationship with hsa-circ-0046159 and ATPase sarcoplasmic/endoplasmic reticulum Ca^2+^ transporting 2 (ATP2A2) and plays an important regulatory role in CTEPH (11). The expression of miR-93-5p is downregulated in CTEPH and correlates with the severity of the disease (12). The hsa-miR-106b-5p is involved in the development of CTEPH *via* targeting matrix metalloproteinase 2 (MMP2) (13). In the recent years, molecular studies on CTEPH have gradually increased; however, its specific pathogenesis remains unclear. In addition, the correlation between lncRNAs and mRNAs and the regulation mechanism of ceRNA on CTEPH have not been reported.

Thus, in this study, first, we sequenced the lncRNAs and mRNAs expression profile data of patients with CTEPH and then screened out differentially expressed lncRNAs (DElncRNAs) and differentially expressed mRNAs (DEmRNAs). Subsequently, we performed the functional enrichment, lncRNA-mRNA co-expression analysis, and ceRNA (lncRNA-miRNA-mRNA) network construction. Finally, we identified the alterations in some molecular markers and signaling pathways in CTEPH.

## Materials and methods

### Study design

This study complied with the Declaration of Helsinki and was approved by the ethics committee (IRB no. 2017-24). The informed consents were obtained from all the participants. From January 2020 to May 2022, 26 patients with CTEPH (12 females, 53.4 ± 11.2 years) and 35 healthy volunteers (13 females, 48.8 ± 9.5 years) were prospectively included in this study. CTEPH was diagnosed by right heart catheterization (RHC) with pulmonary ventilation and perfusion scan and computed tomography pulmonary angiography (CTPA). All healthy volunteers came from our health examination center with the normal lung and liver function, routine blood and urine tests, thyroid hormone levels, and normal electrocardiography. Patients with CTEPH who underwent pulmonary endarterectomy (PEA) were included. Patients who underwent balloon pulmonary angioplasty (BPA) were excluded. Patients without complete data from RHC or patients who underwent PEA before obtaining blood samples also were excluded. Participants with other types of pulmonary hypertension, cigarette-smoking, chronic obstructive pulmonary disease, obstructive sleep apnea syndrome, asthma, infection, interstitial lung disease, connective tissue disease, endocrine diseases, hypertension, diabetes, coronary heart disease, cardiomyopathy, congenital heart disease, malignant tumor, hepatitis and liver cirrhosis, nephritis, and renal failure were all excluded.

The whole blood samples of patients with CTEPH were obtained from the right internal jugular vein during RHC before PEA. The whole blood samples of healthy volunteers were obtained from the right internal jugular vein. The blood samples were flash frozen for later testing. All blood samples were randomly grouped into 2 groups. Group 1, sequencing group (5 females, 51.9 ± 11.2 years) including 7 blood samples from patients with CTEPH and 5 blood samples from the healthy volunteers, used transcriptome sequencing and ceRNA (lncRNA-miRNA-mRNA) network construction. Group 2, validation group (25 females, 50.5 ± 10.8 years) including 19 blood samples from patients with CTEPH and 30 blood samples from the healthy volunteers, used reverse transcription polymerase chain reaction (RT-PCR) validation of genes.

### RNA extraction and library construction

Total RNAs were extracted from whole blood samples of Group 1 using TRIzol^®^ reagent. Nanodrop2000 was used to detect the concentration and purity of the RNA. The integrity of RNA was detected by agarose gel electrophoresis. Agilent2100 was used to determine RNA integrity number (RIN) value. The total amount of RNA required for a single library construction is 5 μg. In addition, the RNA concentration should be ≥200 ng/μl, and the optical density (OD) value should be in 1.8–2.0. DNA fragments in total RNA samples were digested by DNase I, and the reaction products were purified and recovered by magnetic beads. The rRNA was removed from the digested total RNA samples. RNA samples of the previous step were placed in the polymerase chain reaction (PCR) instrument for thermal interruption. Then, one-strand cDNA and two-strand cDNA with dUTP were synthesized in a PCR instrument. Subsequently, terminal repair and connection of the sequencing connector were performed. Finally, PCR amplification was performed to complete the library preparation.

### Sequencing and raw data processing

Agilent 2100 Bioanalyzer and ABI StepOnePlus Real-Time PCR System were used to detect the quality of library. Qualified libraries were sequenced using PE100 strategy on BGIseq platform. The “FASTP” was used for quality control of sequencing data. Specifically, adapter sequence, 5′ segment, 3′ segment, bases with quality <20, and reads with N >10% were trimmed. In transcriptome sequencing data, only the data compared to the reference genome can be used for subsequent analysis. The high-quality sequencing sequences obtained after quality control were compared with the designated reference genome using HISAT2 (https://ccb.jhu.edu/software/hisat2/index.shtml). The reference genome is from Ensembl database, the genome version is GRCh38, and gene annotation information is Ensemble 92. Stringtie (http://ccb.jhu.edu/software/stringtie/) quantifies mRNA and lncRNA expression and standardizes output. Stringtie calculated fragments per kilobase of exon model per million mapped reads (FPKM) value of each gene/transcript in the sample according to the comparison results of HISAT2 software and took this value as the expression level of the gene/transcript in the sample.

### Difference analysis of mRNAs and lncRNAs

First, the original read count was standardized (mainly to correct the sequencing depth). Then, the hypothesis test probability (*p*-value) was calculated by statistical model. Third, multiple hypothesis testing correction (Benjiamini and Hochberg method) (14) was performed to obtain the corrected *p*-value (false discovery rate, FDR). The DESeq2 package in R was used to screen genes with significant differences between different samples (15). *p* < 0.05 and |log2 fold change| (|log2FC|) >1 were used to the differential expression screening criteria of lncRNAs and mRNAs.

### Functional analysis and protein-protein interaction network construction of DEmRNAs

To investigate the distribution and biological pathways involved of DEmRNAs, functional analysis was performed. Gene Ontology (GO) and Kyoto Encyclopedia of Genes and Genomes (KEGG) functional analyses of DEmRNAs were performed using GeneCodis4.0 database (https://genecodis.genyo.es/). The screening criteria were pval_adj <0.05. To further investigate the interaction between DEmRNAs, we constructed a protein-protein interaction (PPI) network. We used string database (https://string-db.org/) to perform protein interaction network analysis for DEmRNAs. The screening criteria was combined score >0.4.

### Analysis co-expression of mRNAs-lncRNA

The functions of lncRNAs are related to their co-expressed protein-coding genes. When there is a positive or negative correlation between the expression of lncRNA and some distant genes, the target gene of lncRNA can be predicted by correlation analysis. To investigate the correlation between DEmRNAs and DElncRNAs, we constructed a co-expression network. In this analysis, Pearson correlation coefficient method was used to analyze the correlation between DElncRNAs and DEmRNAs. The screening criteria were |correlation coefficient| (|r|) ≥0.9, *p* < 0.05.

### Construction of ceRNA (lncRNA-miRNA-mRNA) network

MiRWalk (http://mirwalk.umm.uni-heidelberg.de/interactions/) was used to predict the miRNAs whose target gene is DEmRNAs. Then, the relationship pairs were selected which have been validated in at least two databases (TargetScan, miRDB, MiRTarBase). NPInter (http://bigdata.ibp.ac.cn/npinter4) was used to predict the miRNAs whose target gene is DElncRNAs. Subsequently, the miRNA dataset GSE56914 was downloaded from the GEO database (16). Then, differential expression analysis of miRNAs was performed. The rank sum test was used to analyze the difference in miRNAs expression between healthy control group and CTEPH group. Finally, the intersection of differentially expressed miRNAs, miRNAs targeted by mRNA and miRNAs targeted by lncRNA, was selected.

### RT-PCR validation of IGKV1-8, PMP22, PIK3R6, KCNMB2-AS1, and TCL6

The total RNAs in Group 2 (49 blood samples from 19 patients with CTEPH and 30 healthy volunteers) were extracted by RNAliquid Ultra Speed Whole Blood (liquid sample) Total RNA Extraction Kit. FastQuant cDNA synthesis kit was used for mRNA/lncRNA reverse transcription. SuperReal PreMix Plus (SYBR Green) was used for RT-PCR validation of mRNA/lncRNA. Each experiment was repeated 3 times. GAPDH and ACTB were used as internal control for RT-PCR. All primers are shown in Supplementary Table 1. The 2^−ΔΔCt^ method was used to calculate the relative expression of genes (17).

### Diagnostic analysis

To explore the potential diagnostic value of the identified genes, we performed receiver operating characteristic (ROC) curve analysis using pROC package in R. The diagnostic accuracy of identified genes was evaluated by the area under curve (AUC) values in the ROC curve. If the AUC value is >0.7, it implies that the gene has good diagnostic accuracy (18). The sensitivity and specificity at the cutoffs were determined referring to the previous report (19). ROC diagnostic analysis of the identified genes was performed using the GSE130391 dataset (14 cases of CTEPH and 4 controls). The GSE130391 dataset was obtained from the GEO database.

### Statistical analysis

The DESeq2 package in R was used to screen DEmRNAs and DElncRNA, and the screening criteria were *p* < 0.05, |log2FC| >1. GeneCodis4.0 database was used for functional analysis. The screening criteria were pval_adj <0.05. The Pearson correlation coefficient method was used to analyze the correlation between DElncRNAs and DEmRNAs. The screening criteria were |r| ≥0.9, *P* < 0.05. For the RT-PCR, *t*-test was used to evaluate the statistical significance among the patients with CTEPH and healthy controls.

## Results

### Clinical information

Table 1 shows the clinical data of patients with CTEPH and healthy controls. There was no significant difference of gender (χ^2^ = 0.166, *P* = 0.684) and age (*t* = 1.638, *P* = 0.098) between patients with CTEPH and healthy controls. Gender (χ^2^ = 0.003, *p* = 0.975) and age (*t* = 0.408, *p* = 0.684) between Group 1 (sequencing group) and Group 2 (validation group) were similar. The mean pulmonary artery pressure (mPAP), pulmonary vascular resistance (PVR), pulmonary arterial wedge pressure (PAWP), cardiac output (CO), 6-min walk distance, and N-terminal pro-B type natriuretic peptide (NT-proBNP) of patients with CTEPH, respectively, were 37.85±9.35 mmHg, 681.7dyn.sec.cm^−5^ (IQR, 426.8–974 dyn.sec.cm^−5^), 11.08 ± 2.23 mmHg, 3.5 ± 1.06 L/min, 438.59 ± 102.2 m, and 403 pg/ml (IQR,177–764 pg/ml). NT-proBNP of patients with CTEPH significantly increased in comparison with the healthy volunteers (median = 35 pg/ml, IQR, 21–53 pg/ml) (U = 60.5, *p* < 0.001).

**Table 1:** Clinical information of patients with CTEPH and healthy volunteers.

**Gender**	**Age** (**years**)	**Group**	**Diagnosis**	**Systolic PAP (mmHg)**	**Diastolic PAP (mmHg)**	**mean PAP (mmHg)**	**PVR** (**dyn.sec.cm-5**)	**PAWP (mmHg)**	**CO (L/min)**	**NT-proBNP** (**pg/ml**)	**6MWD (m)**	**Treatment**
Male	36	1	CTEPH	56	16	28	272.4	12	5.87	168	618	PEA
Female	61	1	CTEPH	76	16	36	1068.8	4	2.5	1,312	340	PEA
Male	65	1	CTEPH	80	30	47	1420.4	9	2.14	1,099	300	PEA
Male	44	1	CTEPH	94	34	54	1072	12	3.13	1,106	271	PEA
Female	54	1	CTEPH	86	22	43	535.6	14	4.33	615	477	PEA
Male	57	1	CTEPH	48	22	31	674.4	10	2.45	712	450	PEA
Female	39	1	CTEPH	63	27	39	566.6	13	3.67	677	400	PEA
Female	69	2	CTEPH	76	27	45	1072.2	11	2.39	168	450	PEA
Female	31	2	CTEPH	52	12	25	819.9	9	2.44	34	618	PEA
Female	56	2	CTEPH	71	28	42	954.5	12	2.51	554	477	PEA
Male	61	2	CTEPH	82	28	46	1224.8	8	2.57	4,857	245	PEA
Male	61	2	CTEPH	55	20	32	306.4	12	4.44	25	-	PEA
Male	37	2	CTEPH	38	14	25	207.1	13	4.25	47	550	PEA
Male	43	2	CTEPH	48	16	27	583.2	12	2.47	921	-	PEA
Female	45	2	CTEPH	73	19	37	1032.7	10	2.71	410	450	PEA
Male	60	2	CTEPH	82	19	40	503.4	12	4.45	396	-	PEA
Male	58	2	CTEPH	48	16	27	308.5	9	4.67	180	446	PEA
Male	66	2	CTEPH	75	29	44	850.1	13	2.92	248	360	PEA
Female	46	2	CTEPH	74	19	37	747.7	10	2.89	685	-	PEA
Female	67	2	CTEPH	96	23	47	829.5	13	3.28	387	488	PEA
Female	61	2	CTEPH	52	19	31	456.6	12	3.33	323	464	PEA
Male	64	2	CTEPH	34	18	26	290.1	13	3.58	1,392	534	PEA
Female	32	2	CTEPH	65	21	45	563.4	14	5.24	360	513	PEA
Female	46	2	CTEPH	95	45	61	810.6	9	5.13	301	406	PEA
Male	67	2	CTEPH	60	18	33	337.5	12	4.74	50	310	PEA
Male	63	2	CTEPH	75	16	36	689	10	3.02	605	482	PEA
Female	61	1	normal	-	-	-	-	-	-	10	-	-
Female	63	1	Normal	-	-	-	-	-	-	12	-	-
Male	62	1	Normal	-	-	-	-	-	-	21	-	-
Male	36	1	Normal	-	-	-	-	-	-	17	-	-
Male	45	1	Normal	-	-	-	-	-	-	10	-	-
Female	38	2	Normal	-	-	-	-	-	-	24	-	-
Female	51	2	Normal	-	-	-	-	-	-	33	-	-
Male	35	2	Normal	-	-	-	-	-	-	53	-	-
Male	45	2	Normal	-	-	-	-	-	-	37	-	-
Male	58	2	Normal	-	-	-	-	-	-	46	-	-
Female	48	2	Normal	-	-	-	-	-	-	57	-	-
Female	50	2	Normal	-	-	-	-	-	-	46	-	-
Male	58	2	Normal	-	-	-	-	-	-	74	-	-
Female	54	2	Normal	-	-	-	-	-	-	45	-	-
Male	39	2	Normal	-	-	-	-	-	-	65	-	-
Male	37	2	Normal	-	-	-	-	-	-	44	-	-
Male	53	2	Normal	-	-	-	-	-	-	53	-	-
Male	69	2	Normal	-	-	-	-	-	-	27	-	-
Female	59	2	Normal	-	-	-	-	-	-	45	-	-
Female	44	2	Normal	-	-	-	-	-	-	62	-	-
Male	39	2	Normal	-	-	-	-	-	-	54	-	-
Male	47	2	Normal	-	-	-	-	-	-	45	-	-
Female	48	2	Normal	-	-	-	-	-	-	32	-	-
Female	41	2	Normal	-	-	-	-	-	-	72	-	-
Male	50	2	Normal	-	-	-	-	-	-	34	-	-
Female	38	2	Normal	-	-	-	-	-	-	52	-	-
Male	62	2	Normal	-	-	-	-	-	-	10	-	-
Male	52	2	Normal	-	-	-	-	-	-	16	-	-
Female	36	2	Normal	-	-	-	-	-	-	22	-	-
Female	52	2	Normal	-	-	-	-	-	-	65	-	-
Female	39	2	Normal	-	-	-	-	-	-	12	-	-
Female	34	2	Normal	-	-	-	-	-	-	24	-	-
Male	52	2	Normal	-	-	-	-	-	-	35	-	-
Female	56	2	Normal	-	-	-	-	-	-	24	-	-
Female	57	2	Normal	-	-	-	-	-	-	16	-	-

*Group: 1, transcriptome sequencing group; 2, validation group; CTEPH, chronic thromboembolic pulmonary hypertension; PAP, pulmonary arterial pressure; PVR, pulmonary vascular resistance; PAWP, pulmonary arterial wedge pressure; CO, cardiac output; NT-proBNP, N-terminal pro-B type natriuretic peptide; 6MWD, 6-minute walk test; PEA, Pulmonary endarterectomy*.

### DEmRNAs and DElncRNAs

**Figure 1:**
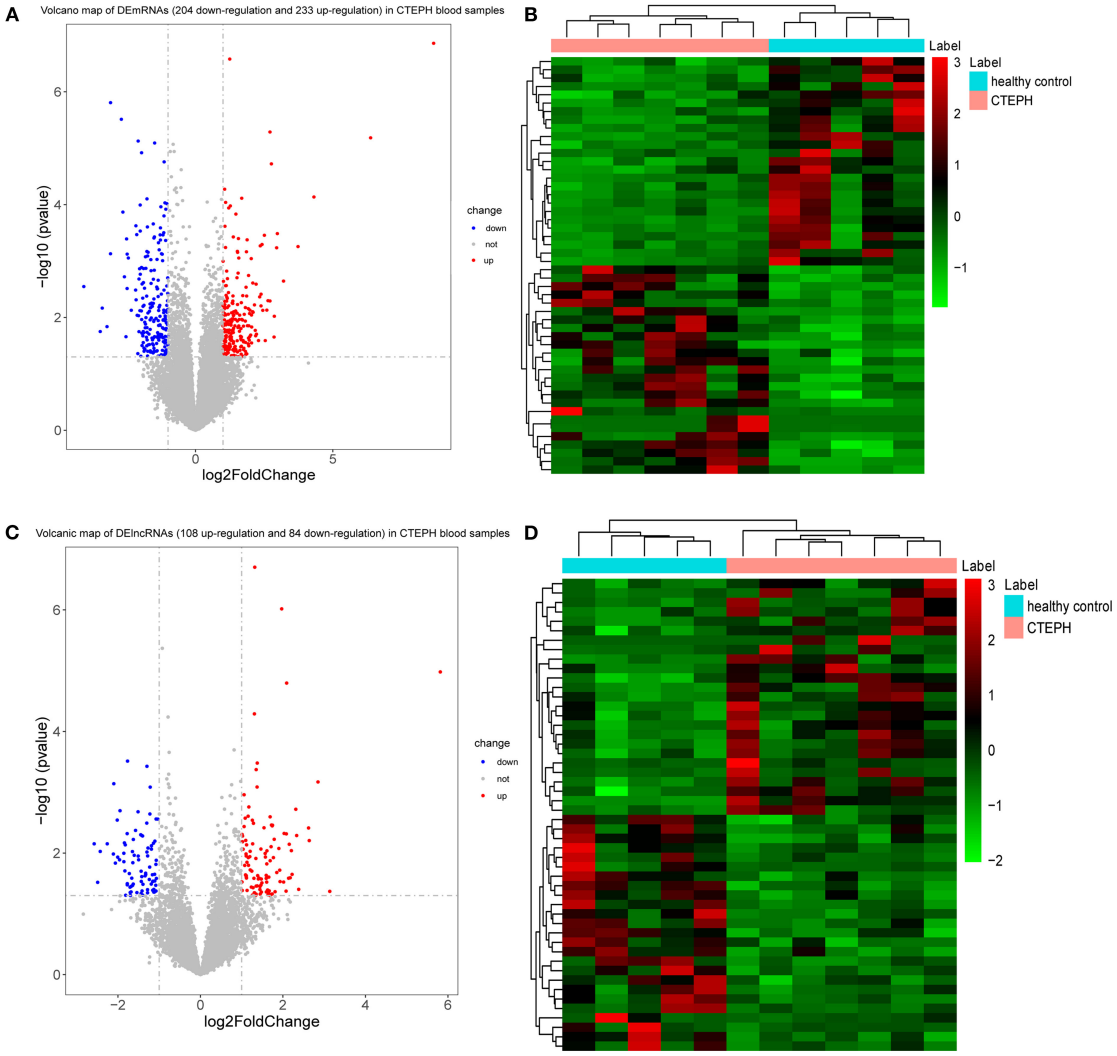
DEmRNAs and DElncRNAs analysis. **(A)** Volcano map of DEmRNAs. X-axis and Y-axis presents -log10 (*p*-value) and log2fold change, respectively. Blue and red colors represent downregulation and upregulation, respectively. **(B)** Heat map of top 50 DEmRNAs. The clustering is constructed using the full chain method together with the Euclidean distance. Red indicates above the reference channel (high expression genes). Green indicates below the reference channel (low expression genes). **(C)** Volcano map of DElncRNAs. X-axis and Y-axis presents -log10 (*p*-value) and log2fold change, respectively. Blue and red colors represent downregulation and upregulation, respectively. **(D)** Heat map of top 50 DElncRNAs. The clustering is constructed using the full chain method together with the Euclidean distance. Red indicates above the reference channel (high expression genes). Green indicates below the reference channel (low expression genes). *p*-Value < 0.05 and |log2 fold change| (|log2FC|) >1 were used to the differential expression screening criteria of lncRNAs and mRNAs.

A total of 437 DEmRNAs were obtained (*p* < 0.05, |log2FC| >1). Among them, 233 expression levels were upregulated, and 204 expression levels were downregulated. Figures 1A,B demonstrate the volcano map of DEmRNAs and the heat map of top 50 DEmRNAs. Table 2 shows the top 10 up-DEmRNAs including WDR90, FAM86B2, CD300LD, MCRIP2, DERPC, PLXNA4, ELN, IGKV1-8, PGAM1P7, and SH3RF3. The top 10 down-DEmRNAs were AP000867.4, RNASE3, ARHGAP8, CEBPE, AC103792.1, TMEM144, SLC29A1, ADGRG5, PIK3R6, and PMP22 (Table 2).

**Table 2:** Top 10 up/down-regulated DEmRNAs.

**ID**	**Gene**	**Log2FoldChange**	***P*** **-value**	**Up/down**
ENSG00000284625.1	AP000867.4	−3.093661715	1.56E-06	Down
ENSG00000169397.3	RNASE3	−2.699640041	3.07E-06	Down
ENSG00000241484.9	ARHGAP8	−2.087374614	7.46E-06	Down
ENSG00000092067.5	CEBPE	−1.488030552	8.07E-06	Down
ENSG00000271350.1	AC103792.1	−1.963230562	1.20E-05	Down
ENSG00000164124.11	TMEM144	−1.14179294	1.74E-05	Down
ENSG00000112759.19	SLC29A1	−1.76693469	7.89E-05	Down
ENSG00000159618.16	ADGRG5	−1.112153433	9.29E-05	Down
ENSG00000276231.5	PIK3R6	−1.043173531	9.60E-05	Down
ENSG00000109099.15	PMP22	−2.013409978	0.000101403	Down
ENSG00000161996.19	WDR90	8.670989479	1.38E-07	Up
ENSG00000145002.12	FAM86B2	1.246973302	2.63E-07	Up
ENSG00000204345.1	CD300LD	2.711903774	5.15E-06	Up
ENSG00000172366.20	MCRIP2	6.376510103	6.54E-06	Up
ENSG00000286140.1	DERPC	2.76345347	1.89E-05	Up
ENSG00000221866.9	PLXNA4	1.060943242	5.33E-05	Up
ENSG00000049540.17	ELN	4.312537384	7.31E-05	Up
ENSG00000240671.4	IGKV1-8	1.683117657	7.70E-05	Up
ENSG00000213997.3	PGAM1P7	1.097032909	9.12E-05	Up
ENSG00000172985.11	SH3RF3	1.267830035	0.000105253	Up

A total of 192 DElncRNAs were obtained (*p* < 0.05, |log2FC| >1). Among them, 108 expression levels were upregulated, and 84 expression levels were downregulated. Figures 1C,D demonstrate the volcano map of DElncRNAs and the heat map of top 50 DElncRNAs. Table 3 shows that LINC00544, AC011484.1, AL022341.1, AC010624.3, AL133384.2, AC103831.1, TCL6, AL157895.2, CCDC144NL-AS1, and AC015912.3 were the top 10 up-DElncRNAs, whereas AC099521.3, AP004608.1, AC020910.2, AC023480.1, LINC01149, U91324.1, AC020895.2, AC004593.1, Z99774.1, and AL157394.1 were the top 10 down-DElncRNAs.

**Table 3:** Top 10 up/down-regulated DElncRNAs.

**ID**	**Gene**	**Log2FoldChange**	***P*** **-value**	**Up/down**
ENSG00000279693.1	AC099521.3	−1.767251736	0.000307162	Down
ENSG00000255545.8	AP004608.1	−1.296864033	0.000374053	Down
ENSG00000268683.1	AC020910.2	−2.100253881	0.000723104	Down
ENSG00000287720.1	AC023480.1	−1.217946334	0.000822968	Down
ENSG00000230174.1	LINC01149	−1.949650534	0.002010516	Down
ENSG00000229740.3	U91324.1	−1.512600273	0.002093572	Down
ENSG00000280091.1	AC020895.2	−1.22547933	0.00227565	Down
ENSG00000285081.1	AC004593.1	−1.075258675	0.002754953	Down
ENSG00000206028.1	Z99774.1	−1.033921668	0.002763125	Down
ENSG00000261438.1	AL157394.1	−2.017209437	0.002867163	Down
ENSG00000122043.11	LINC00544	1.316885707	1.99E-07	Up
ENSG00000204850.4	AC011484.1	1.969416643	9.61E-07	Up
ENSG00000228201.1	AL022341.1	5.815310148	1.05E-05	Up
ENSG00000269091.6	AC010624.3	2.090027053	1.60E-05	Up
ENSG00000279796.1	AL133384.2	1.308093881	5.13E-05	Up
ENSG00000253924.2	AC103831.1	1.377436801	0.000330773	Up
ENSG00000187621.14	TCL6	1.35151838	0.00042378	Up
ENSG00000233968.7	AL157895.2	2.849787772	0.000676503	Up
ENSG00000233098.9	CCDC144NL-AS1	1.373207385	0.000819346	Up
ENSG00000274213.1	AC015912.3	1.058484014	0.001099103	Up

### Functional enrichment analysis and PPI network construction of DEmRNAs

**Figure 2:**
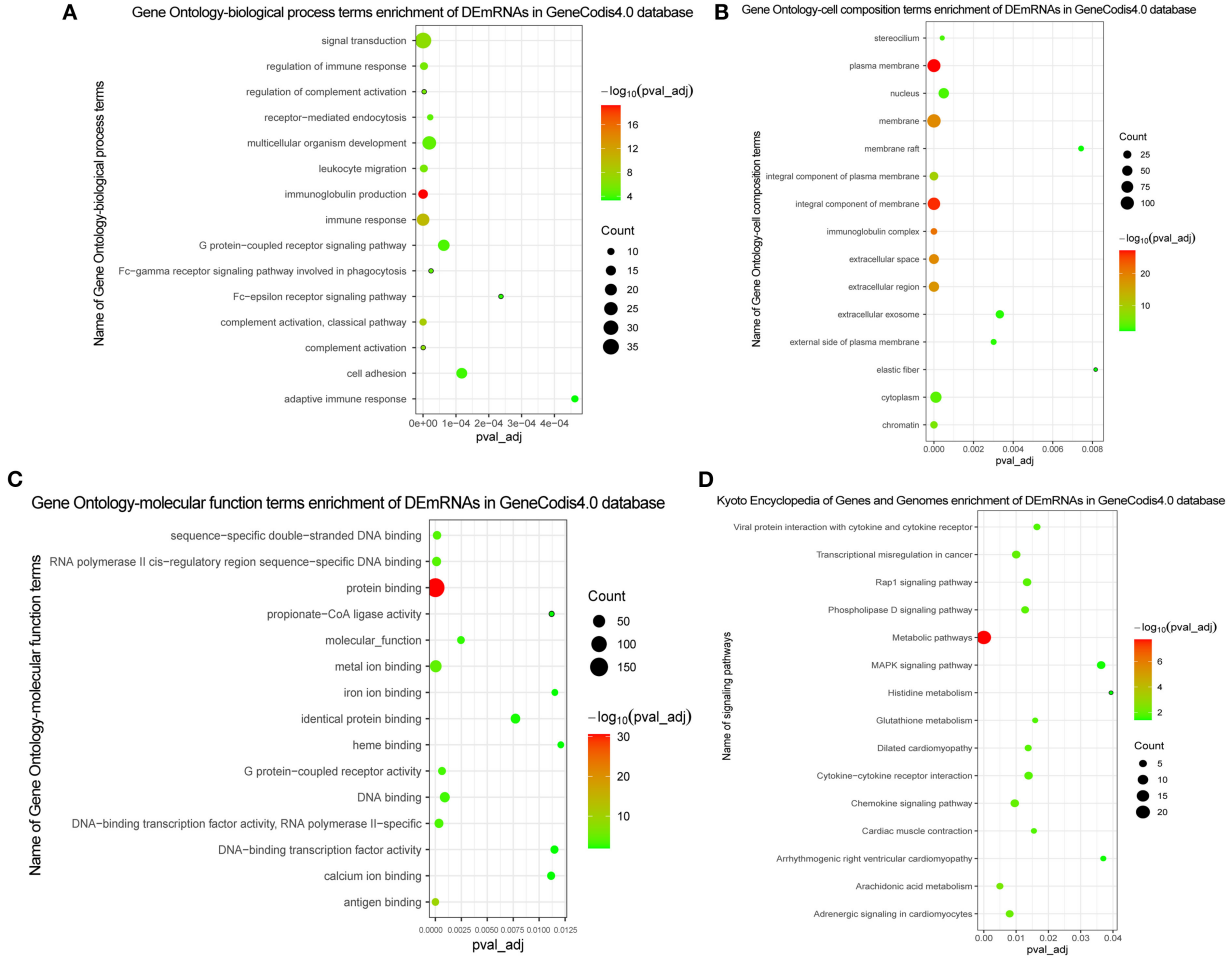
GO and KEGG functional enrichment of DEmRNAs. **(A)** In GO function enrichment analysis, top 15 significantly enriched biological process of DEmRNAs; **(B)** In GO function enrichment analysis, top 15 significantly enriched cell composition of DEmRNAs; **(C)** In GO function enrichment analysis, top 15 significantly enriched molecular function of DEmRNAs; **(D)** In KEGG function enrichment analysis, top 15 significantly enriched signaling pathways of DEmRNAs. The bubble on the graph represents the size of the pval_adj, the redder the more significant. The screening criteria were pval_adj <0.05.

**Figure 3:**
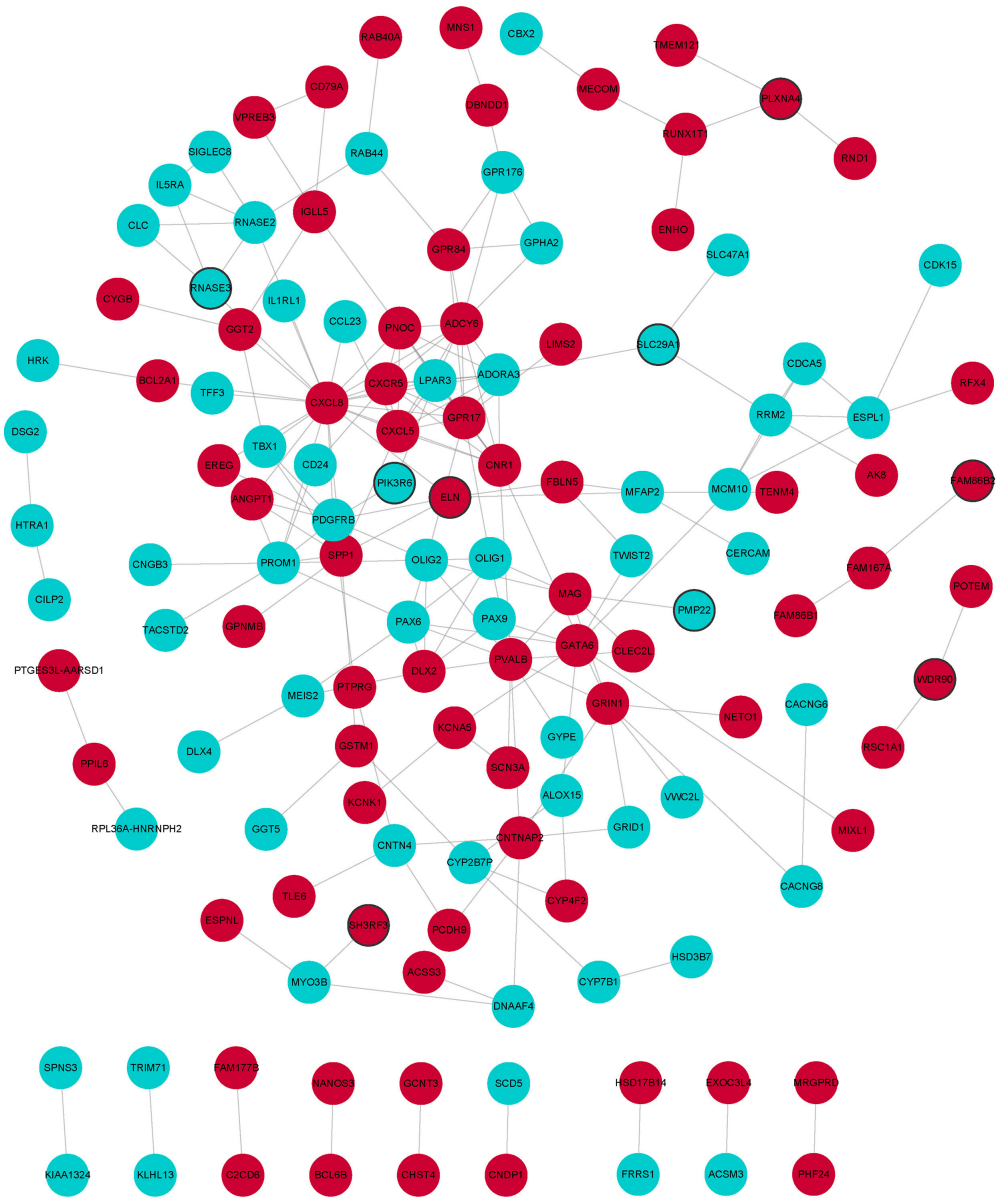
PPI network of DEmRNAs. Red and wathet represent upregulation and downregulation of DEmRNAs. Black borders represent the top 10 up-/downregulated DEmRNAs.

In GO analysis, DEmRNAs were involved in a variety of biological pathways. The biological process, cell composition, and molecular function of the top 15 are shown in Figures 2A–C. In KEGG analysis, DEmRNAs were mainly involved in metabolic pathways, arachidonic acid metabolism, and MAPK signaling pathway (Figure 2D). PPI analysis was performed on 437 DEmRNAs using string database. According to the screening criteria combined_score >0.4, 205 pairs of interacting DEmRNAs were obtained. In the PPI network, 135 nodes and 205 edges were included (Figure 3). Genes with higher degree were CXCL8 (degree = 22, up), GPR17 (degree = 12, up), PVALB (degree = 11, up), ADCY6 (degree = 11, up), CNR1 (degree = 10, up), and CXCL5 (degree = 10, up).

### Analysis co-expression of lncRNA-mRNA

**Figure 4:**
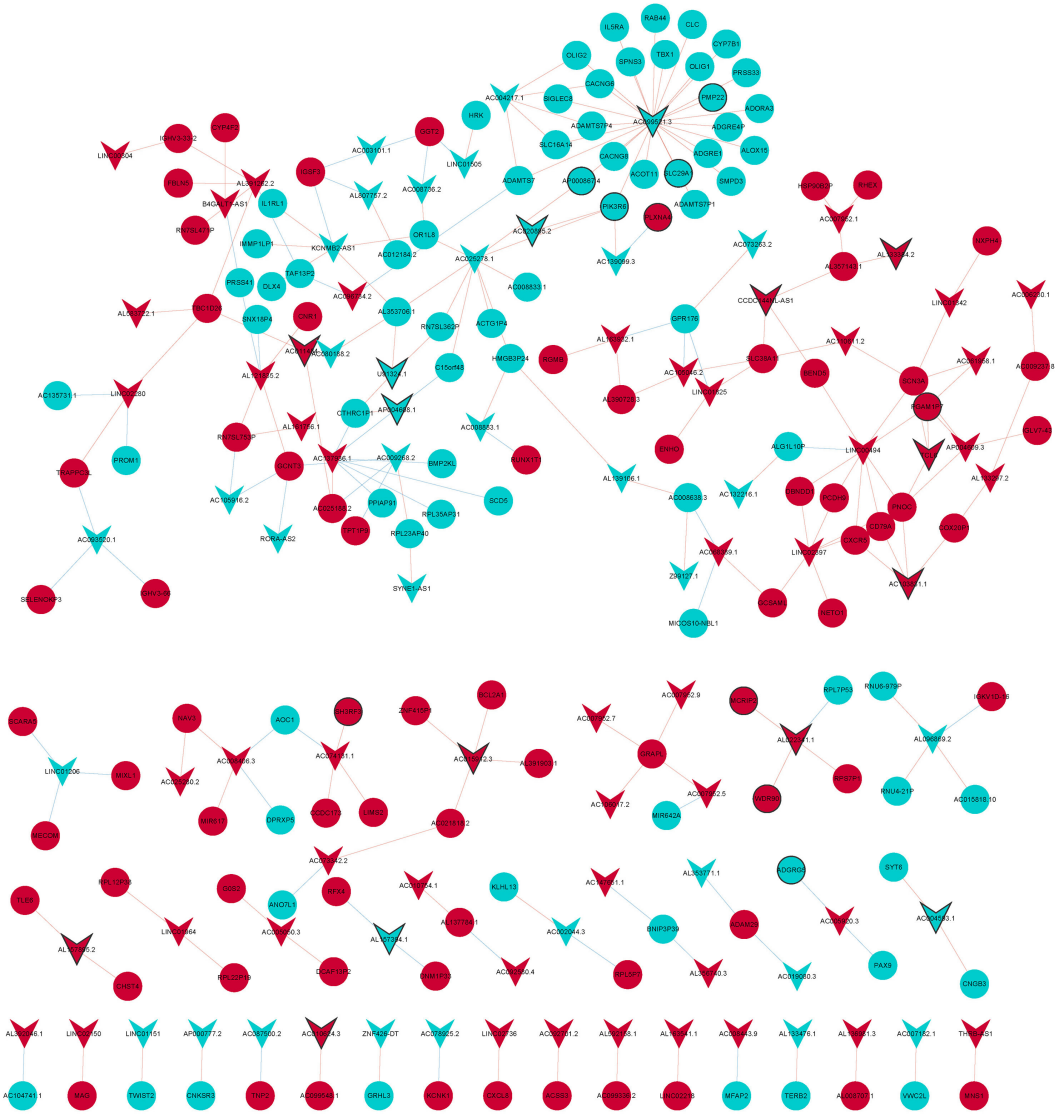
LncRNA-mRNA co-expression network diagram. Circles and V-shape represent DEmRNAs and DElncRNAs, respectively. Red and wathet represent upregulation and downregulation, respectively. Black borders represent the top 10 up-/downregulated DEmRNAs or DElncRNAs.

**Figure 5:**
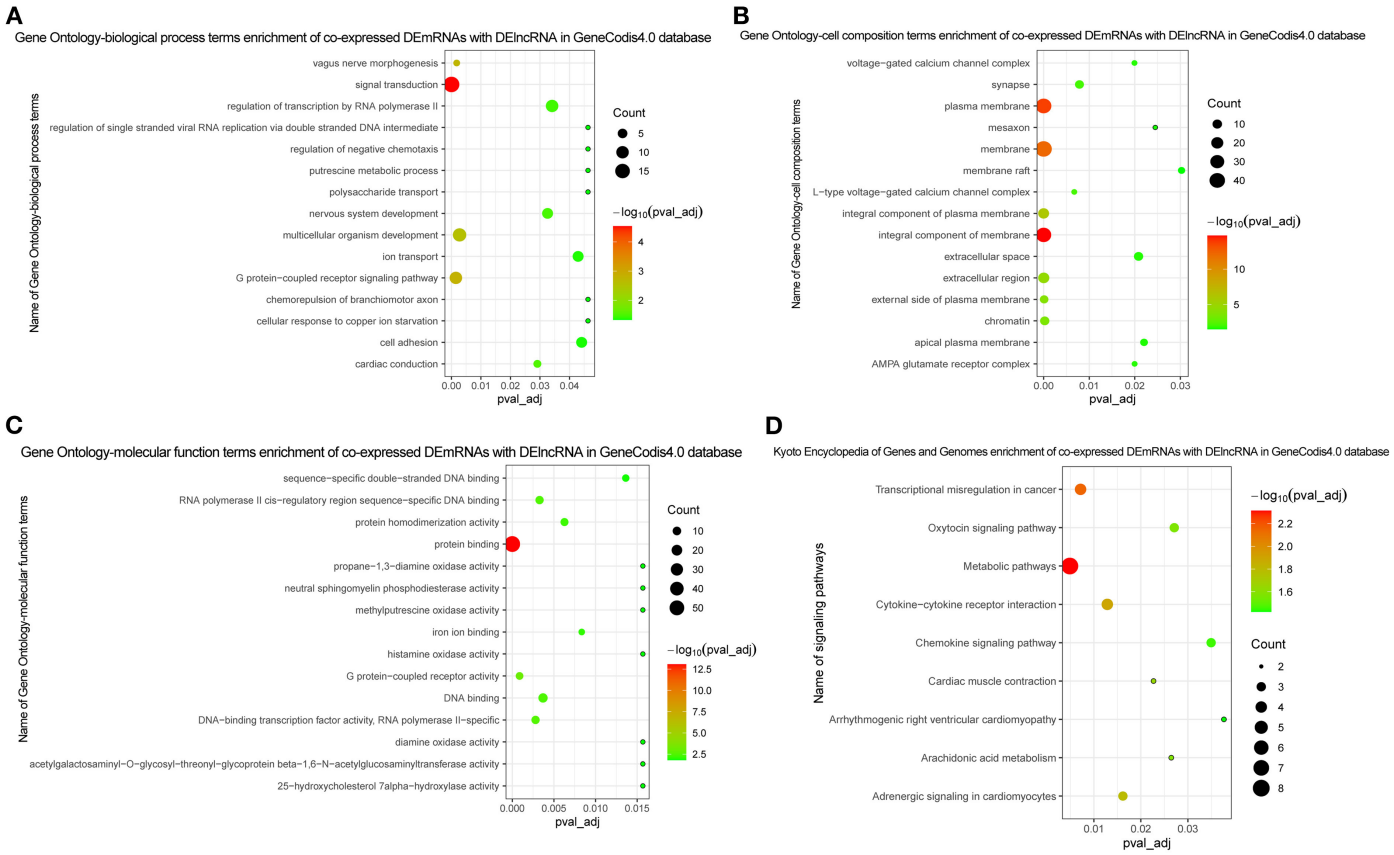
Functional enrichment of co-expressed DEmRNAs with DElncRNA. **(A)** In GO function enrichment analysis, top 15 significantly enriched biological process of co-expressed DEmRNAs; **(B)** In GO function enrichment analysis, top 15 significantly enriched cell composition of co-expressed DEmRNAs; **(C)** In GO function enrichment analysis, top 15 significantly enriched molecular function of co-expressed DEmRNAs; **(D)** In KEGG function enrichment analysis, all enriched signaling pathways of co-expressed DEmRNAs. The bubble on the graph represents the size of the pval_adj, the more red the more significant. The screening criteria was pval_adj <0.05.

The Pearson correlation coefficient method was used to analyze the co-expression of lncRNA-mRNA. According to the screening criteria |r|≥0.9, *p* < 0.05, a total of 232 lncRNA-mRNA relationship pairs were obtained (Figure 4). After that, we performed functional enrichment of the co-expressed DEmRNAs. In GO analysis, DEmRNAs were involved in a variety of biological pathways. The biological process, cell composition, and molecular function of the top 15 are shown in Figures 5A–C. In KEGG analysis, DEmRNAs were mainly involved in metabolic pathways, chemokine signaling pathway, cytokine–cytokine receptor interaction, and arachidonic acid metabolism signaling pathways (Figure 5D).

### Construction of ceRNA network

**Figure 6:**
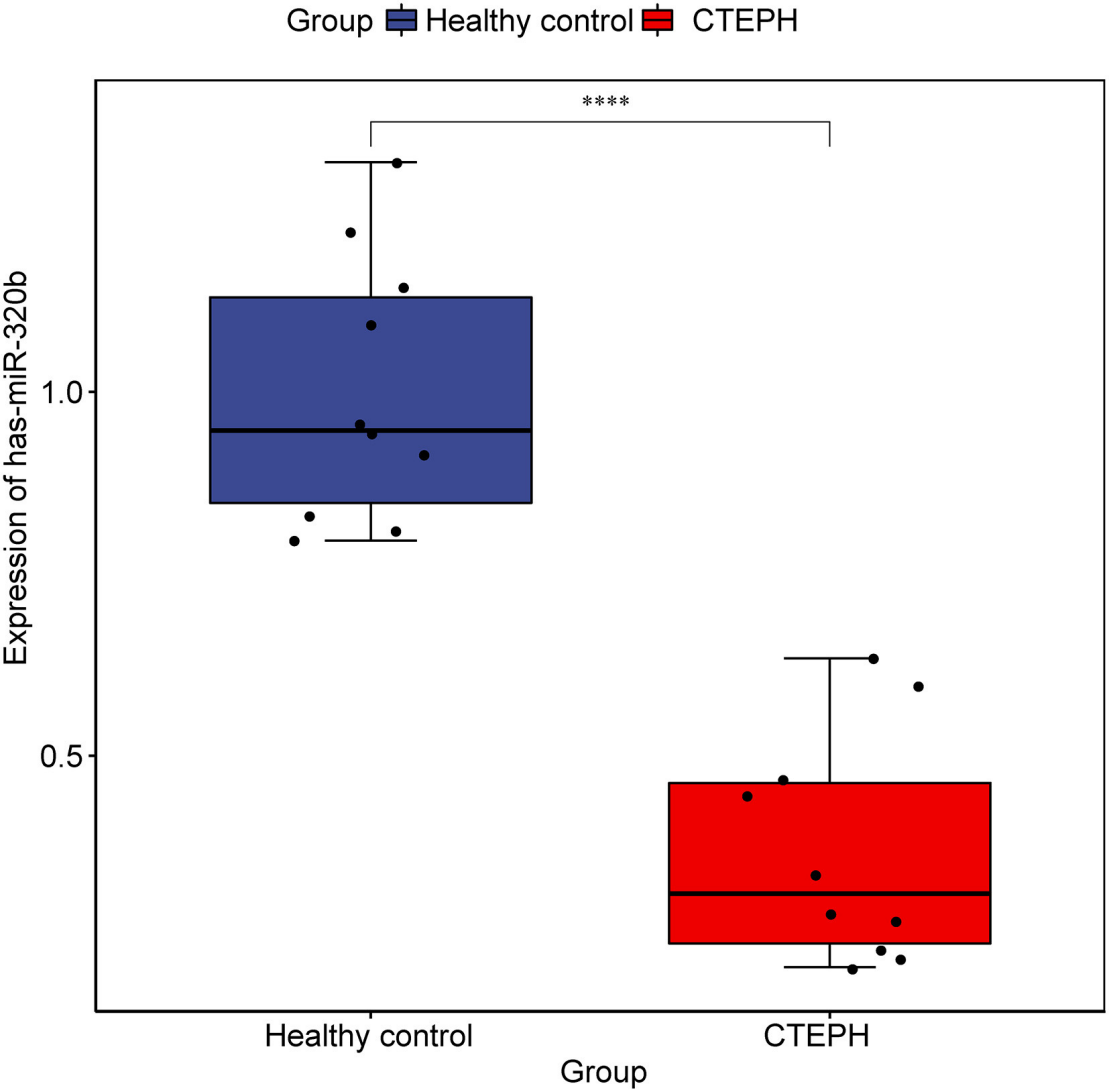
Expression of hsa-miR-320b in samples from dataset GSE56914. The rank sum test was used to analyze the difference of hsa-miR-320b expression between healthy control group and CTEPH group. **** represents *p* < 0.0001. *p* < 0.05 was considered statistically significant.

We obtained 749 mRNA-miRNA targeting relationship pairs *via* miRWalk prediction. Then, 749 lncRNA-miRNA targeting relationship pairs were obtained *via* NPInter prediction. Subsequently, differential expression analysis of miRNAs in GSE56914 dataset was performed. Finally, the intersection of differentially expressed miRNAs, miRNAs targeted by mRNA and miRNAs targeted by lncRNA, was selected. Surprisingly, only one miRNA (hsa-miR-320b) was obtained and its expression was downregulated (Figure 6). The regulatory relationship involved in hsa-miR-320b was SOBP (upregulated)-hsa-miR-320b (downregulated)-LINC00472 (upregulated).

### Diagnostic analysis of genes

**Figure 7:**
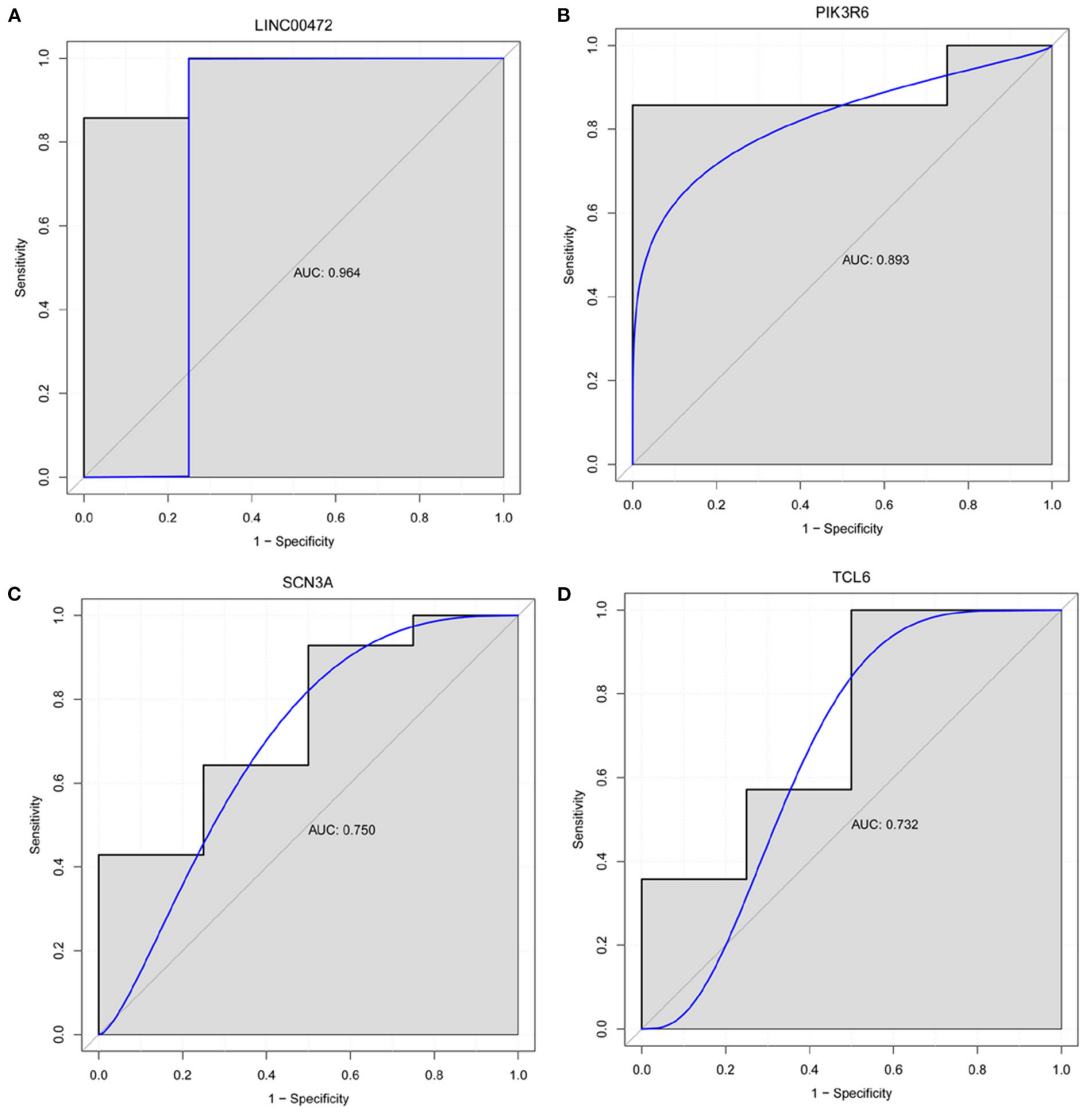
Diagnostic analysis of LINC00472 **(A)**, PIK3R6 **(B)**, SCN3A **(C)** and TCL6 **(D)**. In ROC curve analysis, the AUC value of LINC00472, PIK3R6, SCN3A, and TCL6 was >0.7. It is indicated that LINC00472, PIK3R6, SCN3A, and TCL6 may act as the potential gene markers in CTEPH.

We selected the genes that differentially express up-/downregulation of top 10 or involved in lncRNA-mRNA co-expression/ceRNA network or have been reported to be associated with pulmonary hypertension for diagnostic analysis in GSE130391 dataset. In ROC curve analysis, only the AUC values of LINC00472, PIK3R6, SCN3A, and TCL6 were >0.7, which, respectively, were 0.964, 0.893, 0.750, and 0.732 (Figure 7). It is indicated that LINC00472, PIK3R6, SCN3A, and TCL6 may act as the potential gene markers in CTEPH.

### RT-PCR validation

**Figure 8:**
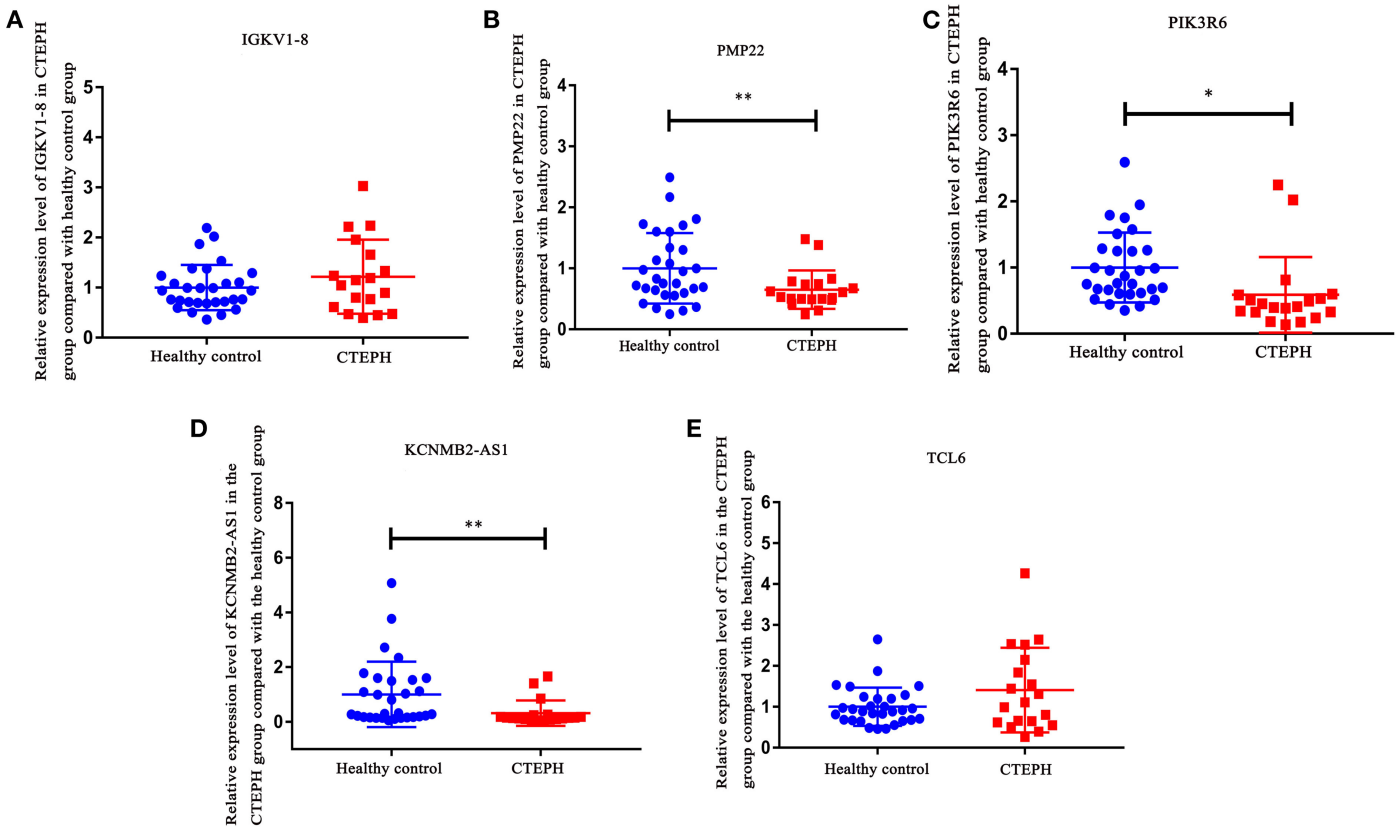
RT-PCR validation of IGKV1-8 **(A)**, PMP22 **(B)**, PIK3R6 **(C)**, KCNMB2-AS1 **(D)**, and TCL6 **(E)** in blood samples. In comparison with the healthy controls, the expression levels of IGKV1-8 and TCL6 in CTEPH group were upregulated trend and PMP22, PIK3R6, and KCNMB2-AS1 were downregulated trend in CTEPH group. Statistical analysis of RT-PCR results showed that the changes in PMP22, PIK3R6, and KCNMB2-AS1 expression levels were statistically significant (*p* < 0.05). The *t*-test was used to evaluate the statistical significance among the patients with CTEPH and healthy controls. * represents *p* < 0.05; ** represents *p* < 0.01. *p* < 0.05 was considered statistically significant.

IGKV1-8, PMP22, PIK3R6, KCNMB2-AS1, and TCL6 were selected for RT-PCR verification (Figure 8). In comparison with the healthy controls, the expression levels of IGKV1-8 and TCL6 in CTEPH group were upregulated trend and PMP22, PIK3R6, and KCNMB2-AS1 were downregulated trend, which was consistent with the analysis results of transcriptome data. Statistical analysis of RT-PCR results showed that the changes of PMP22, PIK3R6, and KCNMB2-AS1 expression levels were statistically significant (*p* < 0.05).

Furthermore, the expression of IGKV1-8, PMP22, PIK3R6, KCNMB2-AS1, and TCL6 in male and female patients with CTEPH was analyzed separately. Compared with the control group, the expression levels of IGKV1-8 and TCL6 in male and female patients with CTEPH were all upregulated trend and PMP22, PIK3R6 and KCNMB2-AS1 were all downregulated trend (Supplementary Figure 1). This indicates that the expression of IGKV1-8, PMP22, PIK3R6, KCNMB2-AS1 and TCL6 in CTEPH may not be sex-specific.

## Discussion

Chronic thromboembolic pulmonary hypertension is an uncommon but severe sequela of venous thromboembolism. Diagnosis remains challenging due to unspecific early clinical symptoms and overlap with other cardiopulmonary diseases (3). Mass spectrometry and bioinformatic analysis of specimens from thromboembolic material with clear thrombus after PEA in 5 patients with CTEPH indicated differentially mediated proteins and abnormal pathways associated with endothelial dysfunction (20). Therefore, it is of great theoretical and clinical significance to study the genetic background of CTEPH. Miao et al. (11) obtained peripheral blood samples from 5 CTEPH and 5 healthy controls for miRNAs and circRNAs differential expression analysis, and the results showed that a large number of miRNAs and circRNAs expressions were abnormal in CTEPH. Although this study laid a foundation for further research on the molecular mechanism of CTEPH, the analysis of lncRNAs was lacking. So far, we have not found relevant literature reports on lncRNAs sequencing of CTEPH. Therefore, lncRNA and mRNA were analyzed in our study. In addition, ROC analysis found that LINC00472, PIK3R6, SCN3A, and TCL6 may be the potential diagnostic biomarkers for CTEPH, which may contribute to the early diagnosis and management of CTEPH.

In this study, we assessed the differences in molecular markers and signaling pathways of patients with CTEPH, in comparison with normal healthy people, through transcriptome sequencing and bioinformatics analysis. However, only one regulation pathway of SOBP-hsa-miR-320b-LINC00472 was found through ceRNA network construction. In comparison with healthy people, sine oculis-binding protein homolog (SOBP) in patients with CTEPH was upregulated. As far as we know, this is the first study to show that SOBP is upregulated express in CTEPH and SOBP may play a potential regulatory role in the pathogenesis of CTEPH. Hsa-miR-320b was found downregulated in CTEPH. Hsa-miR-320b is positively correlated with the survival of chronic obstructive pulmonary disease (COPD), and its expression level can be used to predict the survival of COPD (21). Hsa-miR-320b is low expressed in carotid atherosclerosis (22). Hsa-miR-320b also inhibits lung cancer angiogenesis and tumor growth by inhibiting the hepatocyte nuclear factor 4 gamma (HNF4G) and insulin-like growth factor 2 mRNA-binding protein 2 (IGF2BP2) expression (23). In this study, hsa-miR-320b was the only one miRNA screened and downregulated in CTEPH. Moreover, we found that LINC00472 was upregulated and LINC00472 has better predictive value (AUC = 0.964) in CTEPH. LINC00472 expression level is upregulated in atherosclerotic coronary artery tissue in comparison with normal coronary artery samples (24). Moreover, LINC00472 can regulate the migration and proliferation of vascular smooth muscle cells by regulating miR-149-3p (24). However, the underlying molecular mechanism of SOBP-hsa-miR-320b-LINC00472 needs further verification.

In addition to the above 3 genes screened by ceRNA, we also found PLXNA4, ELN, IGKV1-8, PMP22, CXCL8, SCN3A, and PIK3R6. They were DEmRNAs that differentially express up-/downregulation of top 10 or involved in lncRNA-mRNA co-expression or have been reported to be associated with pulmonary hypertension. The expression level of plexin A4 (PLXNA4) increased significantly in rat pulmonary fibrosis tissue and is highly correlated with the expression level of MRAK081523 (25). PLXNA4 promotes angiogenesis by enhancing vascular endothelial growth factor (VEGF) and bFGF signaling pathways (26). PLXNA4 is a novel candidate gene for pulmonary embolism (PE) in patients with venous thromboembolism (VTE) and plays a role in the process/pathway of thrombosis (27). Previous studies have found that peripheral myelin protein 22 (PMP22) interacts with transforming growth factor-β (TGF-β) (28). TGF-β can induce epithelial–mesenchymal transition (EMT), which had implications for fibrotic lung disease (29). In this study, PLXNA4 and PMP22 were the genes that differentially express up-/downregulation of top 10 and were involved in the co-expression of mRNA-lncRNA. Therefore, we speculated that PLXNA4 and PMP22 may cause the occurrence of CTEPH by affecting vascular formation or reconstruction.

In this study, sodium voltage-gated channel alpha subunit 3 (SCN3A) was upregulated in CTEPH. SCN3A is highly expressed in pulmonary artery smooth muscle cells (PASMC) and is the main α-subunit that forms functional Na^+^ channels in PASMC. Electrical excitability plays an important role in excitation-contractile coupling of the pulmonary vascular system, and it is regulated by transmembrane ion flux in PASMC (30). Therefore, we speculate that SCN3A may play an important regulatory role in the occurrence of CTEPH by regulating the excitation-contraction mechanism of pulmonary vessels. Moreover, SCN3A was also regulated by multiple lncRNAs in CTEPH. Conspicuously, ROC analysis found that the AUC of SCN3A was >0.7. We speculated that SCN3A might be a potential therapeutic target in CTEPH. Therefore, the identification of SCN3A provides an important direction for further research on the molecular mechanism of CTEPH.

In this study, elastin (ELN) was significantly abnormal in CTEPH. ELN plays a key role in lung development. The ELN haploinsufficiency may adversely affect pulmonary vessels. Lacking one elastin allele (Eln^+/−^) neonatal pulmonary capillary deficiency may cause pulmonary hypertension in adulthood (31). In cardiovascular disease, ELN is also associated with high blood pressure and stiff blood vessels (32). We also found that immunoglobulin kappa variable 1-8 (IGKV1-8) was significantly abnormal in CTEPH. IGKV1-8 is highly expressed in pulmonary light chain deposition disease and is associated with diffuse cystic patterns (33). Therefore, we speculate that ELN and IGKV1-8 may play an essential regulatory role in the pathological mechanism of CTEPH.

Compared with the normal controls, the expression of C-X-C motif chemokine ligand 8 (CXCL8) in idiopathic pulmonary arterial hypertension (IPAH) is increased and was associated with an increased risk of IPAH and unfavorable clinical features (34). CXCL8 is also involved in the fibroplasia of canine idiopathic pulmonary fibrosis (35). In this study, although CXCL8 was not the gene that differentially express up-/downregulation of top 10, the degree is equal to 22 in PPI. This indicates that the abnormal expression of CXCL8 in CTEPH may inhibit or activate the functions of other genes, thus playing a regulatory mechanism in the development of CTEPH.

Previous studies have found that phosphoinositide-3-kinase regulatory subunit 6 (PIK3R6) plays an important role in cardiac fibrosis (36). In this study, functional enrichment analysis found that PIK3R6 was involved in the chemokine signaling pathway. Moreover, ROC analysis found that the AUC value of PIK3R6 was >0.7. The occurrence and development of pulmonary hypertension were related to the abnormal expression of a variety of chemokines and chemokine receptors in pulmonary vessels. In addition, some chemokines are differentially regulated in the over-stressed right ventricle (37–40). Therefore, we speculated that PIK3R6 may play an important role in the regulation of CTEPH through the regulation of chemokine signaling pathway.

We also found AC099521.3, LINC01149, KCNMB2-AS1, and TCL6. They were DElncRNAs that differentially express up-/downregulation of top 10 or involved in lncRNA-mRNA co-expression. LINC01149 is significantly associated with childhood asthma, but asthma is a risk factor for COPD (41). Therefore, we speculated that LINC01149 was also a risk factor for CTEPH and plays an important regulatory role in the progression of CTEPH. So far, we have not found any research on TCL6, KCNMB2-AS1, and AC099521.3 in pulmonary hypertension. In this article, it may be the first discovery that TCL6, KCNMB2-AS1, and AC099521.3 are abnormally expressed in CTEPH. In addition, we found that TCL6 has better predictive value (AUC = 0.732) in CTEPH. The discoveries of TCL6, KCNMB2-AS1, and AC099521.3 provide new ideas for future studies of CTEPH research.

In this study, except chemokine signaling pathway, we found that the DEmRNAs were also significantly enriched in metabolic pathways, arachidonic acid metabolism, and MAPK signaling pathway. The metabolic pathway determines the formation of vessels (42, 43). Inhibition of mitochondrial glucose oxidation and enhanced glycolysis is an important feature of pulmonary arterial hypertension metabolic dysfunction that may drive the proliferative phenotype of pulmonary vascular cells (44). Arachidonic acid metabolites play an important role in regulating PH in neonates' pigs with chronic hypoxia (45). Cyclooxygenase-2 (COX-2) is a key enzyme in arachidonic acid metabolism pathway, and its deficiency can lead to severe PH under hypoxic conditions (46). MAPK signaling pathway plays an important role in PH due to left heart disease (47). Previous studies have found that activation of the MAPK signal can regulate pulmonary vascular tension and plays an important role in hypoxia-induced PH development (48, 49). Therefore, we speculate that metabolic pathways, arachidonic acid metabolism, and MAPK signaling pathway play the important roles during the development of CTEPH (50). The identification of metabolic pathways, arachidonic acid metabolism, and MAPK signaling pathway provides regulatory mechanisms research direction for the future.

Although the above findings open new avenues of molecular research about CTEPH, there are some limitations in this study. First, the sample size is relatively small, so it is necessary to expand the sample for further validation study. Second, the specific molecular mechanism of the genes identified in CTEPH is not yet clear, especially the only SOBP-hsa-miR-320b-LINC00472 regulatory pathway. Therefore, a lot of further work is definitely necessary before they are applied routinely to the clinical practice for the evaluation of CTPEH. Moreover, although the expression of IGKV1-8, PMP22, PIK3R6, KCNMB2-AS1, and TCL6 in CTEPH may not be sex-specific, whether patients with CTEPH have specific profile in gender needs to be further studied by expanding the sample.

## Conclusion

Our study is the first study to screen DEmRNAs and DElncRNAs of patients with CTEPH. The research not only significantly enriched in chemokine signaling pathway, metabolic pathways, arachidonic acid metabolism, and MAPK signaling pathway, but also indicated that LINC00472, PIK3R6, SCN3A, and TCL6 may act as the potential gene markers in CTEPH. The alteration in genes and signaling pathways was found through transcriptome sequencing and bioinformatic analysis, which provides a potential direction for future studies on the molecular mechanism of CTEPH. In the future, it might be possible to find potential basis for the prediction and assessment of CTEPH.

## Data availability statement

The datasets presented in this study can be found in online repositories. The names of the repository/repositories and accession number(s) can be found at: Gene Expression Omnibus, accession no. GSE188938.

## Ethics statement

The studies involving human participants were reviewed and approved by the Ethics Committee of China-Japan Friendship Hospital. The patients/participants provided their written informed consent to participate in this study. This study complied with the Declaration of Helsinki and was approved by the Ethics Committee of China-Japan Friendship Hospital (IRB no. 2022-KY-048).

## Author contributions

Conceptualization: ML and YZ. Data curation: WX, MD, XM, XS, DW, and XL. Formal analysis: WX, MD, and DW. Funding acquisition: ML. Investigation: WX, MD, XT, DW, SZ, YZ, and XL. Methodology: XT and SZ. Supervision: ML and DW. Writing—original draft: WX and MD. Writing—review and editing: All authors. All authors contributed to the article and approved the submitted version.

## Funding

This work was supported by Chinese Academy of Medical Sciences Innovation Fund for Medical Sciences (2021-I2M-1-049), National High Level Hospital Clinical Research Funding and Elite Medical Professionals Project of China-Japan Friendship Hospital (2022-NHLHCRF-LX-01 and ZRJY2021-BJ02), and the National Natural Science Foundation of China (Grant No. 81871328).

## Conflict of interest

The authors declare that the research was conducted in the absence of any commercial or financial relationships that could be construed as a potential conflict of interest.

## Publisher's note

All claims expressed in this article are solely those of the authors and do not necessarily represent those of their affiliated organizations, or those of the publisher, the editors and the reviewers. Any product that may be evaluated in this article, or claim that may be made by its manufacturer, is not guaranteed or endorsed by the publisher.

## Supplementary Material

Supplementary Figure 1 Expression of IGKV1-8 **(A)**, PMP22 **(B)**, PIK3R6 **(C)**, KCNMB2-AS1 **(D)**, and TCL6 **(E)** in male and female patients with CTEPH. Expression of IGKV1-8 and TCL6 in male and female patients with CTEPH were upregulated trend and PMP22, PIK3R6, and KCNMB2-AS1 were downregulated trend in comparison with healthy controls. ^*^ represents *p* < 0.05; ^**^ represents *p* < 0.01; ^***^ represents *p* < 0.001.

Supplementary Table 1 Primer sequence in the RT-PCR.
