# Supplementary figures and images for: The alterations in molecular markers and signaling pathways in chronic thromboembolic pulmonary hypertension, a study with transcriptome sequencing and bioinformatic analysis
Source: Front Cardiovasc Med. 2022 Jul 26;9:961305. doi: 10.3389/fcvm.2022.961305 (PMC9362860; doi:10.3389/fcvm.2022.961305)

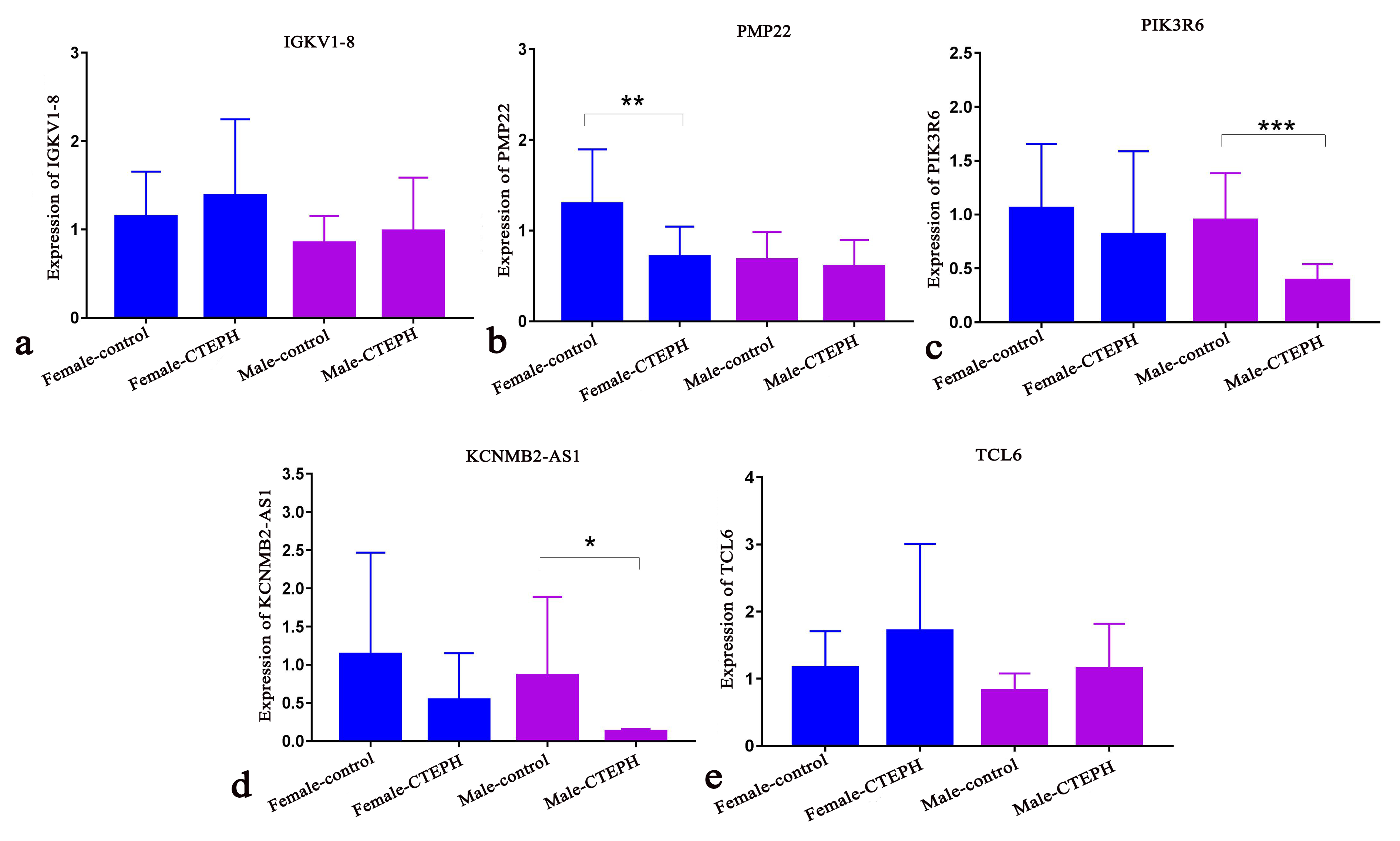

Supplement: Supplementary Figure 1 — Expression of IGKV1-8 (A), PMP22 (B), PIK3R6 (C), KCNMB2-AS1 (D), and TCL6 (E) in male and female patients with CTEPH. Expression of IGKV1-8 and TCL6 in male and female patients with CTEPH were upregulated trend and PMP22, PIK3R6, and KCNMB2-AS1 were downregulated trend in comparison with healthy controls. * represents p < 0.05; ** represents p < 0.01; *** represents p < 0.001. [file Image_1.TIF]
