# Supplementary material for: The alterations in molecular markers and signaling pathways in chronic thromboembolic pulmonary hypertension, a study with transcriptome sequencing and bioinformatic analysis
Source: Front Cardiovasc Med. 2022 Jul 26;9:961305. doi: 10.3389/fcvm.2022.961305 (PMC9362860; doi:10.3389/fcvm.2022.961305)
Supplement: Supplementary Table 1 — Primer sequence in the RT-PCR. [file Table_1.docx]

**Table S1. Primer sequence in the RT-PCR**

| **Primer name** | **Primer sequence (5’ to 3’)** |
| --- | --- |
| GAPDH-F (internal reference) | 5-CTGGGCTACACTGAGCACC-3 |
| GAPDH-R (internal reference) | 5-AAGTGGTCGTTGAGGGCAATG-3 |
| ACTB-F (internal reference) | 5-TCCGCAAAGACCTGTACGC-3 |
| ACTB-R (internal reference) | 5-CTGGAAGGTGGACAGCGAG-3 |
| IGKV1-8-F | 5-GAATTTACTCAGCCCAGTGTGC-3 |
| IGKV1-8-R | 5-GACTCGCCCGACAAGTGATG-3 |
| PMP22-F | 5-CTCGGATTACTCCTACGGTTTCG-3 |
| PMP22-R | 5-TCCGCAAGATCACATAGATGACA-3 |
| PIK3R6-F | 5-CTGGTGTCTGCGTCTGTGT-3 |
| PIK3R6-R | 5-ACGGCGTGGAAATAGTGCT-3 |
| KCNMB2-AS1-F | 5-TGTCAGACTCTCGGGACCAT-3 |
| KCNMB2-AS1-R | 5-GCTTTCTCTCGGTTCCTGCT-3 |
| TCL6-F | 5-GAAGCTGGGGAGGTGAGAGT-3 |
| TCL6-R | 5-GGTCTCCCTCCTTCTGCCTT-3 |
